# Elective orthopedic and cardiopulmonary bypass surgery causes a reduction in serum endostatin levels

**DOI:** 10.1186/s40001-014-0061-9

**Published:** 2014-11-08

**Authors:** Torbjörn Åkerfeldt, Lena Gunningberg, Christine Leo Swenne, Göran Ronquist, Anders Larsson

**Affiliations:** Department of Medical Sciences, Section of Clinical Chemistry, Uppsala University, Uppsala, Sweden; Department of Public Health and Caring Sciences, Uppsala University, Uppsala, Sweden

**Keywords:** Angiogenesis, CRP, ELISA, Endostatin, Elective surgery

## Abstract

**Background:**

Endostatin is an endogenous inhibitor of angiogenesis that inhibits neovascularisation. The aim of the study was to evaluate the effect of elective surgery on endostatin levels.

**Methods:**

Blood samples were collected prior to elective surgery and 4 and 30 days postoperatively in 2 patient groups: orthopedic surgery (n =27) and coronary bypass patients (n =21). Serum endostatin levels were measured by ELISA.

**Results:**

Serum endostatin was significantly reduced 30 days after surgery in comparison with presurgical values in both the orthopedic (*P* =0.03) and cardiopulmonary surgery (*P* =0.04) group.

**Conclusion:**

Serum endostatin is reduced 30 days after surgery. This reduction would favor angiogenesis and wound-healing.

## Background

Collagen XVIII is a basement membrane protein with structural similarities to collagen XV [1,2]. Collagen XVIII contains an anti-angiogenic C-terminal non-collagenous domain known as endostatin [3,4]. Endostatin has a molecular weight of 22 kDa and is an endogenous inhibitor of angiogenesis formed by the proteolytic cleavage of the C-terminal domain [5]. The formation of endostatin is mainly induced by elastase, metalloproteinases (MMP) -3, -7, -9, -13, -14 and -20, and cathepsin L [6-8].

Endostatin plays a role in the local balance of angiogenesis as a potent inhibitor and has been suggested to be of particular importance in the growth and spreading of malignant diseases. Endostatin has been shown to inhibit the growth of several tumors of both human and mouse origin [9-13]. Angiogenesis is also important for physiological wound-healing. Healing of cutaneous wounds in mice may be inhibited by recombinant endostatin treatment [14]. Overexpression of endostatin in keratinocytes has also been shown to delay wound-healing [15]. Endostatin has also been shown to inhibit wound repair of lung epithelial cells [16], and to impair healing of gastric ulcers [17].

The endostatin levels in surgical patients may thus influence the rate of wound-healing. Delayed wound-healing is an important surgical problem and it is associated with increased complications such as infections and also increased mortality [18-21].

The aim of the present study was to investigate the effect of postsurgical inflammation on serum endostatin levels in humans. The patients were treated with elective surgery, and thus the patients had low presurgical inflammatory activities. The operations induce a strong inflammatory response and thus are suitable for investigating the effects of traumatic inflammatory response on serum endostatin levels during the wound-healing period [22]. Results from two surgery groups were compared to strengthen the significance of any findings.

## Methods

### Study population

Elective orthopedic surgery (n =27, 13 males and 14 females) and elective cardiopulmonary bypass surgery (n =21, 18 males and 3 females) patients, at the Uppsala University Hospital were included in the study. Blood sampling was performed prior to surgery and on day 4 and day 30 after surgery. The blood samples were collected in Vacutainer tubes (367815, Becton, Dickinson, Franklin Lakes, NJ, USA) without additives, and after clotting the samples were centrifugated at room temperature and the sera were collected and frozen at −22°C. The study was approved by the local ethical board at Uppsala University (2004:237) and all patients signed an informed consent prior to inclusion in the study.

### C-reactive protein (CRP) assay

Serum CRP (reagent: 6 K2601) was analyzed on an Architect Ci8200 analyzer (Abbott Laboratories, Abbott Park, IL, USA). The CRP assay had a total coefficient of variation (CV) of 0.8% at 8 mg/L and the assay calibrator was traceable to CRM 470.

### Endostatin ELISA

Serum levels of endostatin were analyzed using a commercially available ELISA kit for endostatin (DY1098, R&D Systems, Minneapolis, MN, USA). The assays had a total coefficient of variation (CV) of approximately 6%.

### Statistical calculations

Statistical analysis was performed with Statistica 7.1 (StatSoft, Tulsa, OK, USA). Comparisons between presurgical and postsurgical samples were performed with the Wilcoxon matched pair test. Association between endostatin and CRP was investigated with Spearman rank correlation. Descriptive statistics for the different sampling times were reported as median and IQR (interquartile range). *P* <0.05 was regarded as statistically significant throughout the study.

## Results

### Patient characteristics and CRP values

**Figure 1:**
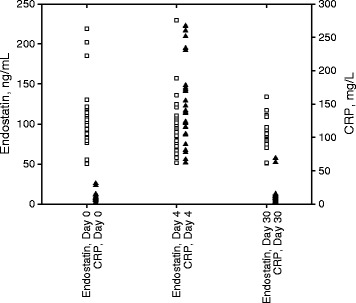
**Endostatin and C-reactive protein (CRP) values in individual patients at day 0, day 4 and day 30 after orthopedic surgery.**

**Figure 2:**
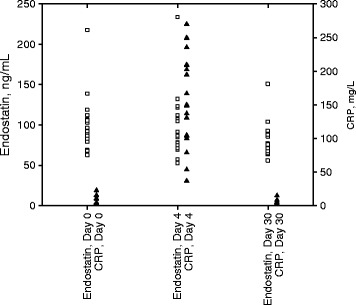
**Endostatin and C-reactive protein (CRP) values in individual patients at day 0, day 4 and day 30 after cardiopulmonary bypass surgery.**

The mean age was 67 years (range 45 to 80 years) for the orthopedic patients and 69 years (range 48 to 84 years) for the cardiopulmonary bypass patients. In the orthopedic group (Figure 1), median CRP value prior to surgery was 1.9 mg/L (IQR 1.2 – 8.7). Four days after surgery the median value was 137.3 mg/L (IQR 104.1 – 178.2) and 30 days after surgery the median value was 5.1 mg/L (IQR 2.1 – 11.2). The corresponding values for the cardiopulmonary surgery group (Figure 2) was: CRP value prior to surgery 3.3 mg/L (IQR 1.0 – 7.6), 4 days after surgery 167.0 mg/L (103.7 – 222.7) and 30 days after surgery 3.4 mg/L (2.0 – 5.6).

### Endostatin values

In the orthopedic group (Figure 1) the presurgical median endostatin value was 98.1 ng/mL (IQR 79.3 – 114.2). Four days after surgery the median value was 92.8 ng/mL (IQR 76.7 – 106.9) (*P* =0.23) and 30 days after surgery the median value was 86.2 ng/mL (IQR 77.7 – 95.3) (*P* =0.03 versus presurgical values). The median endostatin value in the cardiopulmonary surgery group (Figure 2) was 90.0 ng/mL (IQR 81.9 – 105.5). Four days after surgery the median values was 88.1 ng/mL (IQR 77.5 – 112.1) (*P* =0.85) and 30 days after surgery the median value was 76.9 ng/mL (IQR 67.3 – 90.8) (*P* =0.04 versus presurgical values).

### Correlation between endostatin and CRP values

There were significant Spearman rank correlations between endostatin and CRP at day 4 in both the orthopedic (R =0.44, *P* =0.020) and the cardiopulmonary surgery (R =0.54, *P* =0.012) groups (Table 1). The Spearman rank correlations between endostatin and CRP at day 0 and day 30 did not fulfill the preset criteria for significance in this study (*P* <0.05).

**Table 1:** **Spearman rank correlations between C-reactive protein (CRP) and endostatin at day 0, day 4 and day 30 in the orthopedic and the cardiopulmonary surgery groups**

**Orthopedic surgery**	**R**	***P*** **-value**
Day 0	0.35	0.071
Day 4	0.44	0.020
Day 30	0.31	0.15
**Cardiopulmonary surgery**		
Day 0	0.43	0.055
Day 4	0.54	0.012
Day 30	0.29	0.31

## Discussion

Apart from being a breakdown product from collagen XVIII, endostatin is a potent endogenous angiogenesis inhibitor and is regulated in balance with vascular endothelial growth factor (VEGF) [10]. Endostatin has been shown to be a marker of breakdown and remodelling of the extracellular matrix in various diseases [23-25]. Therefore, one plausible explanation for the present associations could be that circulating endostatin also mirrors an increased extracellular remodelling. The surgical procedures result in local tissue damage leading to the initiation of an acute phase reaction and a wound-healing process involving cutaneous, subcutaneous and deeper tissues. The highest CRP values were observed 4 days after surgery. At this sampling time there was a positive Spearman rank correlation between CRP and endostatin concentrations in both patient groups. Thus, during the acute phase there was a positive correlation between inflammatory response and endostatin values. At the same time there was a postoperative endostatin decrease during the postsurgical phase. This indicated that there were several pathways that influenced endostatin concentrations after surgery. One of the influential factors appeared to be the inflammatory response although the surgical process initiated other systems that led to a decrease in endostatin levels.

## Conclusions

The largest difference in serum concentration of endostatin was generated between the samples ‘before surgery’ and ‘day 30’. At day 4 the wound-healing process had just been initiated while it was more active at day 30. The decline in endostatin concentrations favors neovascularisation, which is of importance for wound-healing. Further studies are warranted to explore the different mechanisms that influence endostatin during the wound-healing process.

## Abbreviations

CRP: C-reactive protein
ELISA: Enzyme-linked immunosorbent assay
IQR: Interquartile range
VEGF: Vascular endothelial growth factor
